# Food literacy interventions to improve sustainability in multicultural diets: a scoping review

**DOI:** 10.1017/S1368980026102924

**Published:** 2026-06-22

**Authors:** Sophia Lin, Ebony T. Lewis, Tamanna Jannat Promi, Fiona Ann Christianus, Minh Cuong Duong, Rimante Ronto, Jaimee Hughes, Yuchen Xie, Amanda Duffy, Milena Katz

**Affiliations:** 1 School of Health Sciences, University of New South Wales, Australia; 2 School of Population Health, University of New South Wales, Australia; 3 Department of Health Sciences, Macquarie University, Australia; 4 Social Policy Research Centre, University of New South Wales, Australia; 5 South Western Sydney Local Health District, Australia; 6 NSW Health, Sydney, Australia

**Keywords:** Nutrition, Food literacy, Sustainability, Multicultural, Environment

## Abstract

**Objective:**

This scoping review aimed to identify and map interventions and gaps in evidence that sought to enhance food literacy to support sustainable dietary behaviours among adults from ethnic-minority backgrounds.

**Design:**

A scoping review was conducted following Preferred Reporting Items for Systematic Reviews and Meta-Analyses (PRISMA) guidelines. Studies were included if they targeted food literacy in ethnic-minority adult populations. Nine databases were searched from inception to October 2024.

**Settings:**

Developed economy countries.

**Participants:**

Community-dwelling adult ethnic-minority populations.

**Results:**

Of 3128 records screened, two studies met the inclusion criteria. Both studies involved a retrospective evaluation of outcomes following participation in a community garden programme. Although some sustainability-related outcomes were recorded, none directly integrated food system sustainability education as a primary objective.

**Conclusions:**

This review found very few food literacy interventions that integrate sustainability and are tailored for ethnic-minority communities, highlighting a critical evidence gap. Future health promotion efforts should identify strategies to effectively change food preferences towards nutritious and sustainable choices and how to tailor them for culturally diverse groups to address both health and environmental challenges.

Food production, processing and transportation account for one-third of the global greenhouse gas emissions^(1)^, as well as contributing to significant water and energy consumption, land degradation, environmental waste and biodiversity loss^(2)^. In response, there are global calls for people to change food and eating habits to lessen the environmental impact, such as reducing meat consumption^(3)^, reducing food waste^(4)^ and limiting ultra-processed foods^(5,6)^. These changes benefit both planetary and human health while improving food security^(7)^.

However, environmental sustainability represents only one dimension of sustainable diets. The FAO and WHO^(8)^ define sustainable healthy diets as those with low environmental footprints that are accessible, affordable, safe, equitable and culturally relevant. Consequently, interventions aiming to encourage individuals to make food choices that promote sustainable diets must also address culture, society, community and economic factors. Personal and cultural values are one of the four main factors which influence food purchasing decisions alongside wants and needs of other household members, available time and food budget^(9)^.

Consumer behaviour strongly influences the adoption of sustainable diets, and improving individuals’ food choice skills has been shown to enhance their ability to engage in more sustainable eating practices^(9)^. Although structural and food system interventions are crucial, dietary transitions require individuals to make everyday choices that align with sustainability goals. However, there are insufficient strategies which focus on helping individuals to make more sustainable food choices^(10)^. Strengthening individual knowledge and skills through food literacy interventions offers a promising approach to enhancing sustainable diets, especially as a lack of knowledge currently deters individuals from adopting sustainable diet practices^(11)^.

A recent review confirms that both foundational and critical food knowledge and skills and attitudes/behaviours are essential for developing sustainable food literacy competency, which underpins the ability to achieve sustainable diets^(12)^. Foundational food literacy refers to the basic, essential knowledge about food, nutrition and food systems and is required before progressing to more complex or critical thinking. Food literacy itself has many definitions^(13)^, and Hedin and colleagues^(14)^ highlight the lack of definitions that fully connect food literacy with climate-related food system impacts. The most widely used definition proposes that food literacy is a set of interrelated knowledge, skills and behaviours that enable people to achieve diet quality, dietary resilience and adapt to changes in circumstance^(15)^. These competencies are organised into four domains: planning and managing, selecting, preparing and eating^(15)^.

Critical literacy is defined as the ability to analyse, critique and challenge the norms, rules and practices governing social life^(16)^. It provides the theoretical basis for critical food literacy. Critical food literacy operates at the intersection of food literacy and critical literacy, and represents a higher-order cognitive skill that goes beyond basic food-related knowledge and practices. It requires individuals to find, assess, critique and reflect on problems within the modern food system and to consider how food choices and food environments influence environmental, social and health outcomes. Developing these critical capacities is considered essential for enabling people to understand sustainability concepts such as food waste, climate impacts, and health to support behaviour changes necessary for achieving sustainable diets^(12)^. Hereafter, when the term ‘critical food literacy’ is used in this paper, it refers to the higher order of food literacy skills required for understanding and applying actions that enhance sustainable diets.

There is a bidirectional relationship between healthy diets and the environment^(8)^: reduced consumption of processed food, excessive portion sizes and animal-derived protein are associated with better health outcomes and reduced greenhouse gas emissions, while healthy ecosystems and biodiversity improve human health. Given the increasing importance of ensuring the environmental sustainability of food systems in the modern era, and the bidirectional relationship between healthy diets and environmental sustainability, the development of critical food literacy skills across the lifespan in individuals and populations is vital in improving both human and planetary health. The development and application of food skills must extend beyond food shopping and cooking and incorporate self-reflection on the impact of food choices on both environmental and sociocultural sustainability. However, the importance placed on environmental sustainability and climate action varies across cultural groups^(17–19)^. The influence of these differences on the design and acceptability of critical food literacy interventions in multicultural contexts remains poorly understood.

While there are calls from government^(20)^ and non-government sectors^(3)^ for populations to eat more sustainably, it is not clear how multicultural populations understand or engage in these practices, nor their capacity or desire to do so. Ethnic-minority populations in high-income countries experience long-standing inequities in food environments that directly affect their ability to adopt healthy and sustainable diets. A recent systematic review shows that ethnic minorities are significantly more likely to live in food deserts, with greater exposure to processed food compared with majority populations^(21)^. These structural disparities contribute to poorer diet quality that prevents healthy sustainable diets from being achieved. Moreover, migrants, especially those from lower-income to higher-income countries, often have lower socio-economic status in the new country and are exposed to environments which encourage dietary acculturation^(22)^. Previous literature reviews suggest there are major barriers for people of lower social status in adopting more plant-based eating^(23)^, hinting that migrants in developed economy countries may have less sustainable diets. However, there are differences in specific dietary behaviours between ethnic groups. Meat consumption, which is strongly associated with climate-related impacts, differs between ethnic-minority groups and between first- and second-generation migrants^(24–26)^. In addition, sociocultural factors such as ethnicity, cultural beliefs and food traditions are key determinants of sustainable diet behaviours^(27)^ and may create additional barriers to dietary change in multicultural contexts. Therefore, focusing on ethnic-minority populations is essential for understanding and addressing inequities in the transition towards sustainable dietary patterns in high-income countries.

Sustainability advocacy within food systems often lacks diversity and inclusivity^(18,28)^. Concepts of what constitutes ‘good food’ in sustainability advocacy are shaped largely by Western, middle-class norms, which have marginalised cultural practices and food values from other ethno-cultural groups^(28–30)^ which influence how individuals perceive sustainability and their openness towards programmes which aim to enhance their critical food literacy. There is potential in identifying the strengths of diverse food cultures which may be enhanced to improve sustainable diets. However, research is needed to understand what these factors are and how they can be leveraged to inform culturally responsive food literacy, sustainability and policy initiatives.

Given the conceptual complexity of food literacy, sustainability and cultural tailoring, and the anticipated heterogeneity and limited maturity of the evidence base, a scoping review methodology was selected to examine the extent, characteristics and gaps in this emerging topic area. Therefore, this scoping review aimed to understand the types of interventions used to improve critical food literacy to encourage sustainable food practices in adults from multicultural backgrounds.

## Methods

This scoping review followed the methodology proposed by Arskey and O’Malley^(31)^ and further adapted by the Levac framework^(32)^ and Johanna Briggs Institute^(33)^.

This scoping review includes peer-reviewed literature that describes food literacy programme or policy interventions which indicated they included sustainability-related concepts.

Interventions or programmes that included community-dwelling adults aged 18 years or older were eligible for inclusion. Studies conducted in institutional settings where participants had limited food choices (e.g. hospitals, aged care facilities or prisons) were excluded. Studies were included if they specifically targeted a minority ethnic population group or participants from minority ethnic backgrounds formed most of the study population as a result of general recruitment. In this study, ethnicity is defined as a cultural and social identity that is conceptually distinct from related constructs such as race (a biological categorisation), language (linguistic proficiency) and migration status (country of birth or reason for movement). However, studies often use these factors as proxies for identifying minority ethnic groups. To ensure consistency with the operational definitions used in the literature, we defined ethnic-minority groups as those who did not speak the main national language, were born outside the study country or self-identified as belonging to an ethnic group not predominant in that country^(34)^. Programmes exclusively targeting Indigenous populations were excluded due to their distinct cultural, systemic and historical factors that shape food systems and diet. Indigenous populations have different histories and relationships to the dominant societal group compared to migrant-origin populations. Studies conducted in countries with developing economies were excluded.

The full search strategy is presented in online supplementary material, Supplemental File 1. The inclusion criteria were intentionally narrow to capture interventions that simultaneously addressed food literacy, sustainability and ethnic-minority populations, reflecting real-world public health complexity rather than isolated constructs. All eligible primary research studies including articles, dissertations or reports published in English before October 2024 (no start year limit) were eligible. Both descriptive and analytical observational studies were included. Cross-sectional studies which simply described the prevalence or levels of food literacy skills in a population were excluded. Key search terms covered three main concepts, including (1) food or nutrition, (2) sustainability or environment and (3) migrant or ethnically diverse communities. The Preferred Reporting Items for Systematic Reviews and Meta-Analyses (PRISMA) framework for scoping reviewing was followed. Searches were conducted across the following databases: PubMed, CINAHL, Science Direct, ProQuest, Scopus, Web of Science, Dimensions and Google Scholar. For the latter, the researcher logged out of their Google account to minimise personalised results^(35)^, and the first 100 references sorted by relevance were screened by title and abstract.

Following the search, all identified citations were uploaded to EndNote version 21 (Clarivate Analytics) and then to Covidence screening software (Veritas Health Innovation 2024) for screening, where duplicates were removed. The review team comprised four independent reviewers (SL, TJP, FAC and ETL). At each review stage, two independent reviewers screened citations against the inclusion criteria. Reasons for exclusion at the full-text stage were recorded. Any disagreements were first discussed between the two primary reviewers. If consensus was not achieved, the disagreement was referred to a third reviewer, and when required, a fourth reviewer contributed to final decision-making. The data extracted were Authors, Year of Publication, Country, Study Aim, Population, Sample Size, Intervention Summary, Study Setting and Design, Main Outcomes and Key Findings including literacy level. The data extraction was first piloted using three studies and reviewed independently by all reviewers to ensure consistency. Once alignment was achieved, one reviewer completed data extraction for all included studies.

## Results

**Figure 1.**
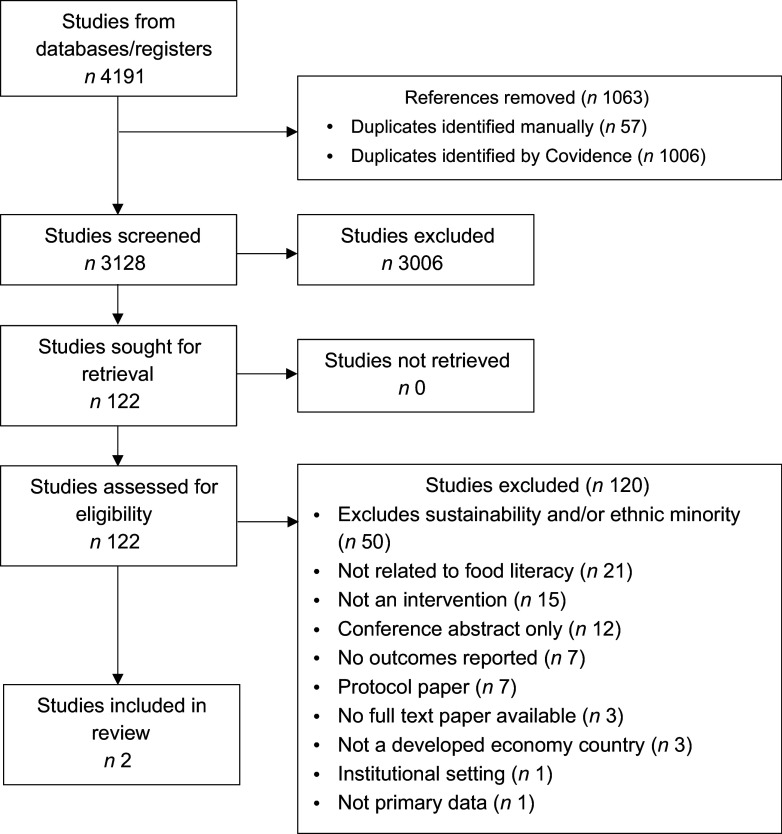
Figure 1 long description. PRISMA flow chart.

The PRISMA flow diagram details the identification and selection of articles (Figure 1). The initial search produced 3128 records. From this, 122 references underwent full text review. Two studies^(36,37)^ met all inclusion criteria: food/nutrition, sustainability/environment and ethnic minority.

Both included studies were conducted in the USA, and both studies involved a retrospective evaluation of participation in a community garden. In the Beavers study^(36)^, approximately two-thirds of the twenty-eight study participants were Black or African American. The study aimed to understand how community gardening impacted on dietary patterns, food security and values and beliefs related to food. The intervention included resources and gardening support, educational classes covering gardening and cooking and social events. Participants reported improved food security as well as improved diet quality with increases in vegetable intake and sustainable dietary practices including a reduction in meat and processed food consumption. Participants indicated that these improvements were driven by increasing their understanding of sustainable food practices including impacts of long food chains and a desire to reduce food waste. In addition, the practice of growing one’s own food resulted in perceptions that the foods tasted better, were fresher, and elicited a greater emotional connection to produce. Mangadu and colleagues’ study^(37)^ was located on the US–Mexico border and aimed to identify best practices in implementing community and school gardens for low-income families. Conducted as a summer camp, the intervention included classes on sustainable gardening, nutrition, healthy cooking and food tasting with garden produce. The outcomes collected from 223 participants were similar to that observed in the Beavers study with improvements in knowledge of portion size, food groups and healthy lifestyle choices, as well as improvements in fruit and vegetable consumption. Participants reported increased caring for the environment and increased knowledge about local foods.

However, although both studies included elements of sustainability as part of programme delivery, and attitudes and behaviours related to food sustainability were reported, they were not programme priorities. There were no identified studies which expressly aimed to change behaviours directly related to food choices known to have significant impacts on planetary health such as meat consumption, food waste and food processing. There were no studies identified that were purposefully designed to enhance individual’s knowledge on the connection between food choices, health and climate change or environmental impacts in the target population.

## Discussion

This scoping review demonstrates an absence of interventions that intentionally integrate sustainability-focused food literacy within ethnic-minority adult populations, indicating that this field remains underdeveloped. Importantly, this finding does not reflect a lack of food literacy research broadly, but rather the lack of interventions that simultaneously address sustainability, food literacy and cultural relevance. The scarcity of evidence identified here represents a major gap with implications for public health nutrition research, equity and climate policy.

Consistent with previous reviews^(38,39)^, most food literacy interventions emphasised food literacy skills such as shopping, cooking, and eating rather than higher-order critical food literacy skills which examined and critiqued food system issues and its relationship to nutrition, health and well-being. Our study extends on previous reviews to determine that among the few examples that incorporated aspects of food system sustainability, these elements were not framed as core study outcomes. Both studies were conducted in the same country which limits understanding of how interventions to enhance critical food literacy skills can be operationalised in diverse sociocultural and structural contexts. Food practices, dietary norms, food environments and food systems differ widely between countries with high migrant populations. Further research is needed in other countries to understand how interventions to enhance critical food literacy skills could be delivered.

Additionally, both identified studies were set in community gardens, and there is a need for further research to understand how critical food literacy skills can be delivered in other settings such as within community organisations, workplaces and schools/universities. There is also a need for research to understand how sustainability can be effectively incorporated into routine nutrition care in clinical settings. At present, some nutrition and dietetic professional organisations have included sustainability as part of practice competencies^(40,41)^, but how this can be implemented effectively, in the context of busy, time-poor clinicians, is understudied. Regardless of setting, culturally tailored interventions are needed to address sustainable diet issues in minority populations.

A recent systematic review of culinary medicine interventions for diverse racial and ethnic-minority populations^(42)^ found that they can be successful in creating behaviour change and improvements in select risk factors. However, this systematic review did not explicitly identify studies with integrated sustainability and included interventions aimed at improving lower-level cognitive and practical skills relating to food and eating which do not enable a person to apply knowledge and skills related to appraise food system issues relating to sustainability^(43,44)^.

Culturally tailored food literacy programmes are linked to positive dietary changes in minority communities^(42,45,46)^. These strategies are also needed to develop critical food literacy skills and sustainability awareness. However, additional challenges may arise due to varying values and priorities related to climate change across cultural groups^(17,19)^, and research is required to understand what these challenges are and how they can be addressed through interventions to increase population adherence to sustainable diets. In the absence of intervention evidence, insights from adjacent literature are drawn to identify priority behaviours and design considerations for future culturally responsive food literacy interventions. The following sections discuss three food-related behaviours with major implications for carbon emissions, meat consumption, food waste and processed food, and the potential barriers to change these behaviours in ethnic-minority populations which sustainable diet interventions must address.

### Reduction of meat consumption

Reducing meat consumption (including red meat, poultry and seafood) is among the most important individual and household actions to reduce dietary carbon footprint^(47)^ and should be a part of food literacy interventions to address sustainable diets. However, meat consumption and attitudes towards plant-based diets vary widely across cultures and require careful consideration in food literacy programme development. There are barriers to achieving more plant-based eating in low socio-economic groups living in high-income countries. Many ethnic-minority groups in high-income countries are of lower socio-economic status, which is associated with higher meat consumption^(23)^. Lowering barriers to consume more plant-based diets can benefit the environment but must be done in ways that are pragmatic and culturally responsive. Recent literature reviews on interventions to reduce meat consumption demonstrate research outside of Europe and North America is lacking^(48)^. Health-framed messaging and social norm strategies, which increase people’s desire to fit in and obtain social approval, are shown to be effective in Western contexts but require tailoring to meet cultural requirements. Social norm messaging is culturally adaptable and may be a useful tool as part of critical food literacy interventions to improve sustainable diets.

In East and Southeast Asian cultures, traditional diets are primarily based on grains and vegetables, with limited meat consumption due to religious, cultural and environmental factors^(30)^. Religious influences such as Buddhism and Taoism in China and Japan have historically restricted meat consumption, while culinary traditions emphasise flavour, aesthetics and variety through smaller portions and shared dining. These features render traditional diets both nutritious and sustainable and should be encouraged to enhance sustainable diets. However, the gradual adoption of Western-style diets due to dietary acculturation post-migration, which increases consumption of meat and processed food, is a challenge. Interventions in this population group must consider the cultural influences on meat consumption to address challenges in promoting sustainable diets. Studies suggest that Chinese consumers, who are one of the largest migrant groups in high-income countries such as Australia^(49)^, UK^(50)^ and the USA^(51)^, prioritise health and sensory satisfaction over environmental or ethical concerns^(52)^, potentially limiting engagement in critical food literacy initiatives. There is also documented resistance to vegetarian advocacy^(53)^ due to clashes with traditional food values. Additionally, Chinese consumers view meat as a symbol of personal social status and that meat-eating is a right^(53)^. The greater the ability to consume meat, the higher the social standing within a community. This viewpoint is reflected in many other communities where socio-economic progress increases with meat consumption^(54)^. Western concepts of vegetarianism are often associated with a social movement and a way to achieve food system sustainability. However, it can be perceived as a form of oppression in countries with colonial histories^(53)^. Western-centric sustainability advocacy, such as the promotion of universal vegetarianism, may be perceived as a form of ideological oppression in communities with colonial histories, as it often overlooks the cultural significance of meat as a hard-won symbol of social and economic progress. Consequently, interventions that do not acknowledge these historical and status-driven contexts risk being viewed as exclusionary or paternalistic, potentially undermining their acceptability among diverse populations. Food literacy interventions to enhance food system sustainability in these communities may need to avoid encouragement of vegetarian diets and emphasise the balance of meat and vegetables in a healthy diet.

Traditional dietary patterns across Latin American cultures are diverse but commonly emphasise legumes, grains (e.g. maize) and plant-based staples, with meat used more sparingly as a complement rather than the central component of meals^(55)^. These dietary patterns are generally aligned with principles of sustainability and nutritional adequacy and may offer a valuable foundation for promoting sustainable diets post-migration. Latino immigrants in the USA report increased consumption of meat and processed foods following migration, often attributed to structural barriers such as limited access to fresh and culturally appropriate foods and the affordability and availability of energy-dense convenience products^(56,57)^. At the same time, recent surveys in the USA indicate that non-Hispanic Black, Hispanic and Asian populations are less likely to consume red meat compared to non-Hispanic White populations, with motivations more strongly linked to environmental and climate-related concerns than to health considerations^(58)^. This suggests a potential openness to sustainability-oriented messaging within Latino communities, although such messaging must be carefully framed.

### Food waste reduction

Reducing food waste is another key strategy for decreasing the environmental impact of the food system. Attitudes strongly shape food waste behaviour. People who display negative attitudes towards food waste, believing it to be harmful, costly and unethical, waste less food^(59)^. Subjective norms also have a strong influence on food waste behaviour, acting both as a barrier and enabler depending on context. In populations with frugality norms, avoiding waste is prioritised while in other populations, creating waste through the preparation of excess food to show hospitality and abundance is expected^(59)^. Evidence from Ethiopia, Brazil and Haiti shows that there are culturally specific ideas about food-related behaviours^(60)^. Behaviours which are sustainable such as eating leftovers to reduce food waste are linked to perceptions of poverty and food insecurity. In addition, behaviours which increase the likelihood of food waste such as having a wide variety of different dishes, having large food stores and hosting social gatherings were associated with wealth and being food secure. Therefore, food literacy interventions aimed at reducing food waste to enhance sustainable diets must consider cultural and social attitudes and how food waste is perceived within communities.

Food waste also increases with larger households^(59)^. In many ethnic-minority communities, multi-generational households are the norm and prioritise eating together at mealtimes, sharing in the same dishes, reflecting values around family and community^(61)^. Ethnographic research in England indicates that food waste is shaped by household preferences, resistance to new or ‘improvised’ meals, and perceptions that fresh food is ‘good’ or ‘proper’ even when unused^(62)^. Evans^(62)^ suggests that the demands of everyday life, which can be more stressful and challenging in multicultural communities due to lower determinants of health, contribute more to food waste practices rather than a lack of knowledge. In multi-generational households, this adds additional complexity in ensuring everyone’s needs and wants are met. Therefore, food literacy interventions should account for local cultural meanings of food and social status when promoting sustainable behaviours.

### Reducing processed food consumption

Consumption of ultra-processed food has significant impacts on both health and environment due to the reliance on resource-intensive commodity crops, excessive packaging and long supply chains^(5,6)^. Migrant and ethnic-minority populations in high-income countries face challenges in reducing their consumption of ultra-processed foods, which has important implications for sustainable diets. Dietary acculturation commonly leads to a shift away from traditional, minimally processed foods towards the more energy-dense, ultra-processed dietary patterns of host countries, with studies showing a 15–20 % increase in processed food intake following migration^(22)^. These shifts are reinforced by structural inequities in food environments. Ethnic-minority populations are 70 % more likely to live in food deserts, with greater exposure to unhealthy food retailers and processed food marketing^(21)^. Reducing processed food consumption in migrant and ethnic-minority communities requires addressing structural and economic barriers as well as food literacy interventions tailored for these communities to enhance sustainable diets.

### Harnessing existing strengths in diverse food cultures

Traditional diets in many ethnic-minority populations are more nutritious and sustainable compared to the average diet in their host country due to a higher consumption of fruit and vegetables and low consumption of meat and highly processed food^(63)^ and are encouraged as a way of achieving healthy sustainable diets^(3)^. There is potential for cultural exchange, blending the best aspects of traditional diets and adapting them for new environments post-migration. Supporting individuals and communities to maintain cultural food practices is an important strategy in achieving both human and planetary health as well as achieving food security. Integration of traditional and modern food cultures can bring opportunities for innovation in enhancing critical food literacy skills and create lasting changes in sustainable food practices. In countries with high proportions of migrants from a wide range of countries such as Australia, Canada, UK and USA, the strengths of traditional multicultural diets remain an untapped potential to drive change. How these strengths can be identified and utilised by public health practitioners and nutrition professionals is not yet clear. In the current drive to act on climate change, diverse voices are often limited, superficial and fail to overcome structural barriers such as economic disparity, historical exclusion and language barriers that enable meaningful engagement and representation in decision-making regarding food systems^(28)^. In addition, many migrants face day-to-day living challenges such as stable housing^(64)^ and finding work or long work hours^(65)^ which may make engagement with food literacy programmes and sustainable diets less of a priority. Therefore, it is imperative that any future dietary interventions to enhance sustainable food choices must be codesigned in partnership with communities which recognised the complex socio-economic, cultural and environmental constraints that shape food practices in migrant communities.

Despite the lack of clear evidence of how critical food literacy skills may be enhanced in multicultural communities, existing evidence from culturally tailored intervention programmes to enhance food literacy skills may be drawn upon. Food represents culture and identity, and for migrants, traditional foods and ways of eating can be a source of comfort and a connection to their roots in a new country^(66)^. To encourage these communities to make dietary changes to improve sustainability, or to manage chronic disease conditions, there can be tensions between maintaining cultural food practices and adopting recommended diets. The Planetary Health Diet developed by the EAT–Lancet Commission has been proposed as a dietary pattern to promote human health while ensuring that global food consumption stays within environmental limits^(47)^. Recent updates to the proposed dietary pattern stress the importance of traditional diets as part of healthy sustainable diets^(3)^ Previous reviews highlight that culturally adapted nutrition interventions lead to significant improvements in diet quality, food security and health outcomes compared to generic programmes^(42)^.

Culturally tailored food literacy programmes acknowledge that food is deeply tied to cultural identity, social norms, values and traditions, and therefore strategies that incorporate these elements are more likely to succeed^(45,46)^. Interventions that ignore these factors risk low engagement and limited effectiveness. For migrant communities, these norms, traditions and values change after arrival in a new country and continue to change with years as an émigré^(67)^. How these norms, traditions and values change will differ for each ethnic minority, depending on their host country due to local economic, political, social and environmental influences. Therefore, tailoring interventions to enhance critical food literacy skills may require adapting or developing new content, ways of delivery and engagement methods that align with norms, traditions and values^(68)^.

### Strengths and limitations

A key strength of this review is its ability to explicitly document the absence of sustainability-integrated food literacy interventions for ethnic-minority adults, providing a clear evidence base to inform future funding priorities and intervention development. This is a significant contribution because most existing food literacy research focuses on food shopping, cooking and eating, neglecting sustainability. The review highlighted critical gaps, such as the lack of culturally tailored programmes that integrate sustainability education which has implications for public health and nutrition practice.

A limitation of this scoping review is the exclusion of developing economy countries, of which many are multi-ethnic with a large range of language, religious and traditional eating practices which inform dietary intake. Broader ethnicity-related terms were intentionally used in this review to avoid excluding articles using specific group labels such as ‘Latino’ or ‘Asian’. Additionally, we focused on the functional status of ‘ethnic minority’ or ‘migrant’ within high-income contexts to capture the shared sociocultural barriers to food literacy. The exclusion criteria may have resulted in the omission of important insights into sustainability practices and culturally embedded food systems. Additionally, there was variability in the outcome measures in the identified studies which makes it difficult to draw strong conclusions about best practices in delivering food system sustainability education to multicultural populations.

### Conclusion

Food literacy interventions are well represented in the general population but frequently lack integrated sustainability education or promote critical food literacy skills. In multicultural populations who often experience higher risks of diet-related disease, the development of these skills has the potential to simultaneously improve nutrition, enhance food security and advance planetary health. Addressing this gap is essential to ensure that sustainability‑oriented dietary transitions do not exacerbate existing health and environmental inequities.

## Supporting information

10.1017/S1368980026102924.sm001 Lin et al. supplementary material Lin et al. supplementary material

## Supplementary material

For supplementary material accompanying this paper visit https://doi.org/10.1017/S1368980026102924

## Figure 1. Long description

The PRISMA flow diagram shows the study selection process. A total of 4191 records were identified through database searching. After removing 1063 duplicates, 3128 records were screened by title and abstract. Of these, 3006 records were excluded. The remaining 122 full-text articles were assessed for eligibility. A total of 120 full-text articles were excluded, with reasons including exclusion of sustainability and/or ethnic minority (n=50), not related to food literacy (n=21), not an intervention (n=15), conference abstract only (n=12), no outcomes reported (n=7), protocol paper (n=7), no full text paper available (n=3), not a developed economy country (n=3), institutional setting (n=1), not primary data (n=1). Two studies met the inclusion criteria and were included in the final review.

Navigate back to Figure 1..

## References

[1] Crippa M , Solazzo E , Guizzardi D et al. (2021) Food systems are responsible for a third of global anthropogenic GHG emissions. Nat Food 2, 198–209. 37117443 10.1038/s43016-021-00225-9

[2] McClelland SC , Haddix JD , Azad S et al. (2023) Quantifying biodiversity impacts of livestock using life-cycle perspectives. Front Ecol Environ 21, 275–281.

[3] Rockstrom J , Thilsted SH , Willett WC et al. (2025) The EAT-Lancet Commission on healthy, sustainable, and just food systems. Lancet 406, 1625–1700. 41046857 10.1016/S0140-6736(25)01201-2

[4] Goossens Y , Wegner A & Schmidt T (2019) Sustainability assessment of food waste prevention measures: review of existing evaluation practices. Front Sustain Food Syst 3, 90.

[5] Leite FHM , Khandpur N , Andrade GC et al. (2022) Ultra-processed foods should be central to global food systems dialogue and action on biodiversity. BMJ Glob Health 7, e008269. 10.1136/bmjgh-2021-008269 PMC8895941 35346976

[6] De-Regil LM , Montez J , Mahy L et al. (2025) Global action on ultra-processed foods: a health, equity, and sustainability imperative. Lancet 406, 2608–2610. 41270765 10.1016/S0140-6736(25)02326-8

[7] Milner J , Hamilton I , Woodcocks J et al. (2020) Health benefits of policies to reduce carbon emissions. BMJ 368, l6758. 32229476 10.1136/bmj.l6758 PMC7190375

[8] FAO & WHO (2019) Sustainable Healthy Diets: Guiding Principles. Food and Agriculture Organization & World Health Organization. Rome. https://www.who.int/publications/i/item/9789241516648 (accessed March 2026).

[9] Fatemi SF , Tehrani H , Khosravi M et al. (2025) Influencing factors of adherence to sustainable diets: a systematic review of behavioral theories. Front Sustain Food Syst 9, 1465622.

[10] Reyes LI , Constantinides SV , Bhandari S et al. (2021) Actions in global nutrition initiatives to promote sustainable healthy diets. Glob Food Secur 31, 100585.

[11] Nichifor B , Zait L & Timiras L (2025) Drivers, barriers, and innovations in sustainable food consumption: a systematic literature review. Sustainability 17, 2233.

[12] McManus S , Pendergast D & Kanasa H (2025) The intersection between food literacy and sustainability: a systematic quantitative literature review. Sustainability 17, 459. doi: 10.3390/su17020459.

[13] Thompson C , Adams J & Vidgen HA (2021) Are we closer to international consensus on the term ‘food literacy’? A systematic scoping review of its use in the academic literature (1998–2019). Nutrients 13, 2006. 34200872 10.3390/nu13062006 PMC8230497

[14] Hedin B , Grönborg L & Johansson G (2022) Food carbon literacy: a definition and framework exemplified by designing and evaluating a digital grocery list for increasing food carbon literacy and changing behavior. Sustainability 14, 12442.

[15] Vidgen HA & Gallegos D (2014) Defining food literacy and its components. Appetite 76, 50–59. 24462490 10.1016/j.appet.2014.01.010

[16] Luke A (2012) Critical literacy: foundational notes. Theory Pract 51, 4–11. doi: 10.1080/00405841.2012.636324.

[17] Ballew M , Maibach E , Kotcher J et al. (2020) Which Racial/Ethnic Groups Care Most About Climate Change? Yale Program on Climate Change Communication. https://climatecommunication.yale.edu/publications/race-and-climate-change (accessed December 2025).

[18] Centre for Climate Change and Social Transformations (CAST) (2024) Ethnicity and UK Climate Perceptions: Why We Need Greater Diversity in Engagement and Research. https://cast.ac.uk/wp-content/uploads/2025/03/CAST-the-centre-for-climate-change-and-social-transformations-report-ethnicity-and-uk-climate-perceptions-why-we-need-greater-diversity-in-engagement-and-research.pdf (accessed December 2025).

[19] Dechezleprêtre A , Fabre A , Kruse T et al. (2025) Fighting climate change: international attitudes toward climate policies. Am Econ Rev 115, 1258–1300.

[20] Australian Department of Health and Aged Care (2023) National Health and Climate Strategy. Canberra: Australian Department of Health and Aged Care.

[21] da Silva Magalhães E , Schattschneider DP , de Vargas EB et al. (2025) Ethnic-racial disparities in food environments: a systematic review and meta-analyses of inequities in food access and availability. J Racial Ethnic Health Disparities. Published online: 10 November 2025. doi: 10.1007/s40615-025-02728-8. 41212385

[22] Varre JV , Dustin M & Van Vliet S (2025) Dietary transformations and health implications in migrant populations: a global perspective. Front Nutr 12, 1623556. 40901297 10.3389/fnut.2025.1623556 PMC12400961

[23] Wiesli TX (2025) Meat consumption among different social groups and specific options for reducing it: a literature review of empirical research. Front Sociol 10, 1547663. 40487004 10.3389/fsoc.2025.1547663 PMC12141276

[24] Meat & Livestock Australia (2025) A Changing Population: Creating New Red Meat Opportunities. MLA News & Events. https://www.mla.com.au/news-and-events/industry-news/a-changing-population-creating-new-red-meat-opportunities/ (accessed March 2026).

[25] United States Department of Agriculture & Economic Research Service (2022) Food Consumption Varies among Racial and Ethnic Groups. USDA ERS Chart Gallery. https://www.ers.usda.gov/data-products/chart-gallery/chart-detail?chartId=103590 (accessed March 2026).

[26] Choi SE & Lee KJ (2023) Ethnic differences in attitudes, beliefs, and patterns of meat consumption among American young women meat eaters. Nutr Res Pract 17, 73–90. 36777805 10.4162/nrp.2023.17.1.73 PMC9884589

[27] Baliwati YF & Rusyda AL (2024) Beyond the plate: how socio-culture and economics drive sustainable diets globally. Jurnal Gizi Indonesia (Indonesian J Nutr) 13, 63–78.

[28] Moore K & Swisher ME (2015) The food movement: growing white privilege, diversity, or empowerment? J Agric Food Syst Commun Dev 5, 115–119.

[29] Jin S , Matsuoka Y , Yue M et al. (2024) Does information about environmental considerations affect Chinese and UK consumers’ purchase intentions for traced foods? A path analysis. Environ Dev Sustain 26, 32287–32318.

[30] Nam KC , Jo C & Lee M (2010) Meat products and consumption culture in the East. Meat Sci 86, 95–102. 20510536 10.1016/j.meatsci.2010.04.026

[31] Arksey H & O’Malley L (2005) Scoping studies: towards a methodological framework. Int J Soc Res Methodol 8, 19–32.

[32] Levac D , Colquhoun H & O’Brien KK (2010) Scoping studies: advancing the methodology. Implement Sci 5, 69. 20854677 10.1186/1748-5908-5-69 PMC2954944

[33] Peters MDJ , Godfrey CM , McInerney P et al. (2015) The Joanna Briggs Institute Reviewers’ Manual 2015: Methodology for JBI Scoping Reviews. Adelaide: The Joanna Briggs Institute.

[34] Chauhan A , Walton M , Manias E et al. (2020) The safety of health care for ethnic minority patients: a systematic review. Int J Equity Health 19, 118. 32641040 10.1186/s12939-020-01223-2 PMC7346414

[35] Bramer WM , Rethlefsen ML , Kleijnen J et al. (2017) Optimal database combinations for literature searches in systematic reviews: a prospective exploratory study. Syst Rev 6, 245. 29208034 10.1186/s13643-017-0644-y PMC5718002

[36] Beavers AW , Atkinson A & Alaimo K (2020) How gardening and a gardener support program in Detroit influence participants’ diet, food security, and food values. J Hunger Environ Nutr 15, 149–169.

[37] Mangadu T , Kelly M , Orezzoli MCE et al. (2017) Best practices for community gardening in a US-Mexico border community. Health Promot Int 32, 1001–1014. 27107021 10.1093/heapro/daw025

[38] O’Brien K , MacDonald-Wicks L & Heaney SE (2024) A scoping review of food literacy interventions. Nutrients 16, 3171. 39339771 10.3390/nu16183171 PMC11435165

[39] Cabezas MF & Nazar G (2023) A scoping review of food and nutrition literacy programs. Health Promot Int 38, 1–18. 10.1093/heapro/daad090 37676303

[40] Dietitians Australia (2021) National Competency Standards for Dietitians in Australia. https://dietitiansaustralia.org.au/sites/default/files/2022-09/National%20Competency%20Standards%20for%20Dietitians%20in%20Australia.pdf (accessed June 2026).

[41] Commission on Dietetic Registration Scope and Standards of Practice Task Force (2024) Revised 2024 Scope and Standards of Practice for the Registered Dietitian Nutritionist. https://www.eatrightpro.org/-/media/files/eatrightpro/practice/scope-and-standards-of-practice/scope-standards-of-practice-2024-for-the-rdn.pdf?rev=2c1952ac84124830a6d9d2cc660b44b3&hash=BE08D3FCB0340C9797C4D583922C37B6 (accessed June 2026).

[42] Brown B , Adediran E , Taylor E et al. (2025) Culinary medicine interventions among racial and ethnic minority and underrepresented populations: a systematic review. Am J Lifestyle Med. Published online: 04 September 2025. doi: 10.1177/15598276251370976. PMC12411487 40918264

[43] Pendergast D , Garvis S & Kanasa H (2011) Insight from the public on home economics and formal food literacy. Fam Consum Sci Res J 39, 415–430.

[44] Slater J (2013) Food literacy education in Manitoba, Canada and Victoria, Australia: a comparative pilot study. Int J Home Econ 13, 16–28.

[45] U.S. Department of Agriculture & U.S. Department of Health and Human Services (2025) Scientific Report of the 2025 Dietary Guidelines Advisory Committee: Part D. Chapter 8 – Culturally Responsive Interventions to Improve Diet. Washington, DC. https://www.dietaryguidelines.gov/2025-advisory-committee-report (accessed June 2026).

[46] Kreuter MW , Lukwago SN , Bucholtz RD et al. (2003) Achieving cultural appropriateness in health promotion programs: targeted and tailored approaches. Health Educ Behav 30, 133–146. 12693519 10.1177/1090198102251021

[47] Willett W , Rockström J , Loken B et al. (2019) Food in the Anthropocene: the EAT–Lancet Commission on healthy diets from sustainable food systems. Lancet 393, 447–492. 30660336 10.1016/S0140-6736(18)31788-4

[48] Kwasny T , Marth S , Dobernig K et al. (2025) Universal truths about reducing meat consumption? Curr Environ Health Rep 12, 33. 40965736 10.1007/s40572-025-00498-3 PMC12446401

[49] Australian Bureau of Statistics (2025) Australia’s Population by Country of Birth. https://www.abs.gov.au/statistics/people/population/australias-population-country-birth/latest-release (accessed June 2026).

[50] Office of National Statistics (2023) The Changing Picture of Long-Term International Migration, England and Wales: Census 2021. https://www.ons.gov.uk/peoplepopulationandcommunity/populationandmigration/internationalmigration/articles/thechangingpictureoflongterminternationalmigrationenglandandwales/census2021#recent-arrivals (accessed June 2026).

[51] Migration Policy Institute (2025) Chinese Immigrants in the United States. https://www.migrationpolicy.org/article/chinese-immigrants-united-states (accessed June 2026).

[52] Baker S , Thompson KE & Palmer-Barnes D (2002) Crisis in the meat industry: a values-based approach to communications strategy. J Mark Commun 8, 19–30.

[53] Zeng G , Chen Z & Zhong S (2024) ‘We Chinese just want meat!’ An analysis of Chinese netizens’ reactions to vegetarian advocacy. Food Qual Prefer 115, 105128.

[54] Sans P & Combris P (2015) World meat consumption patterns: an overview of the last fifty years (1961–2011). Meat Sci 109, 106–111. 26117396 10.1016/j.meatsci.2015.05.012

[55] Valerino-Perea S , Lara-Castor L , Armstrong MEG et al. (2019) Definition of the traditional Mexican diet and its role in health: a systematic review. Nutrients 11, 2803. 31744179 10.3390/nu11112803 PMC6893605

[56] Formagini T , Rodriguez D , Dias J et al. (2025) Reassessing established assumptions of dietary habits in the USA in the context of migration and acculturation: a qualitative study of Latino immigrants. J Racial Ethnic Health Disparities 12, 1333–1343. 10.1007/s40615-024-01967-5 PMC11914326 38668779

[57] Bowen S , Hardison-Moody A , Cordero Oceguera E et al. (2025) Beyond dietary acculturation: how Latina immigrants navigate exclusionary systems to feed their families. Social Prob 72, 819–840.

[58] Sleboda P , de Bruin WB , Baker K et al. (2026) Not eating red meat is associated with reporting the environment and climate change as a top concern: evidence from a national U.S. survey. Humanities Social Sci Commun 13, 295.

[59] Etim E , Choedron KT , Ajai O et al. (2025) Systematic review of factors influencing household food waste behaviour: applying the theory of planned behaviour. WM&R 43, 803–827. 10.1177/0734242X241285423 PMC12106936 39385555

[60] Owens C , Weaver LJ , Kaiser BN et al. (2022) Context matters for food security: multi-sited evidence of shared cultural models of food consumption. Ecol Food Nutr 61, 162–181. 34468242 10.1080/03670244.2021.1969927

[61] Loth KA , Eisenberg ME & Neumark-Sztainer D (2019) The intergenerational transmission of family meal practices. Public Health Nutr 22, 848–856. 30732660 10.1017/S1368980018003920 PMC6715132

[62] Evans D (2011) Blaming the consumer – once again: the social and material contexts of everyday food waste practices in some English households. Crit Public Health 21, 429–440.

[63] Harper K , Batal M & Vatanparast H (2023) Protecting traditional cultural food practices: trends in diet quality and ultra processed food intake among racial/ethnic minority and Indigenous adults in Canada, 2004–2015. Nutrients 15, 3594.37630784

[64] Rana K , Kent JL & Page A (2025) Housing inequalities and health outcomes among migrant and refugee populations in high-income countries: a mixed-methods systematic review. BMC Public Health 25, 1098. 40121396 10.1186/s12889-025-22186-5 PMC11929249

[65] Fasani F (2024) New Approaches to Labour Market Integration of Migrants and Refugees, Publication for the Committee on Employment and Social Affairs, Policy Department for Economic, Scientific and Quality of Life Policies, European Parliament, Luxembourg. https://www.europarl.europa.eu/RegData/etudes/STUD/2024/754232/IPOL_STU(2024)754232_EN.pdf (accessed June 2026).

[66] Dhar S (2024) Balancing Heritage and Health: Significance of Culturally Tailored Dietary Recommendations for Immigrant Communities. The Pursuit: Trending Topics from Michigan Public Health. https://sph.umich.edu/pursuit/2024posts/balancing-heritage-and-health-significance-of-culturally-tailored-dietary-recommendations-for-immigrant-communities.html (accessed December 2025).

[67] Maia AC , Marques MJ , Goes AR et al. (2024) Health literacy strengths and needs among migrant populations: a scoping review. Front Public Health 12, 1415588. 39022410 10.3389/fpubh.2024.1415588 PMC11253791

[68] Livingstone KM , Love P , Mathers JC et al. (2023) Cultural adaptations and tailoring of public health nutrition interventions in Indigenous peoples and ethnic minority groups: opportunities for personalised and precision nutrition. Proc Nutr Soc 82, 478–486. 37334485 10.1017/S002966512300304X

