# Supplementary material for: Food literacy interventions to improve sustainability in multicultural diets: a scoping review
Source: Public Health Nutr. 2026 Jun 22;29(1):e140. doi: 10.1017/S1368980026102924 (PMC13434555; doi:10.1017/S1368980026102924)
Supplement: Supplementary file 1 [file S1368980026102924sup001.docx]

**Supplementary 1: Search strategy**

| Database | Search terms | Results | Limits |
| --- | --- | --- | --- |
| PubMed | (“food litera*” OR “critical food literacy” OR “food choice*” OR “food education” OR “food policy” OR “ecological nutrition” OR “nutrition literacy” OR “food knowledge” OR “food practices” OR “food consumption” OR “food production” OR “food choice*” OR “food knowledge” OR “sustainable food”) AND (environment OR “climate change” OR climate OR “food insecurity” OR “food environments” OR “environmentally sustainable”) AND (migrant OR migrants OR immigrant OR immigrants OR "cultural diversity” OR “sociocultural factors” OR “cultural factors” OR “migrant population” OR “migrant populations” OR “migrant group” OR “migrant groups” OR “immigrant population” OR “immigrant populations” OR “immigrant group” OR “immigrant groups” OR CALD OR “culturally and linguistically diverse” OR ethnic minorit* OR “multicultural” OR “diverse”) | 763 | Studies published in English  Up to 1 Oct 2024 |
| CINAHL | (“food litera*” OR “critical food literacy” OR “food choice*” OR “food education” OR “food policy” OR “ecological nutrition” OR “nutrition literacy” OR “food knowledge” OR “food practices” OR “food consumption” OR “food production” OR “food choice*” OR “food knowledge” OR “sustainable food”) AND (environment OR “climate change” OR climate OR “food insecurity” OR “food environments” OR “environmentally sustainable”) AND (migrant OR migrants OR immigrant OR immigrants OR "cultural diversity” OR “sociocultural factors” OR “cultural factors” OR “migrant population” OR “migrant populations” OR “migrant group” OR “migrant groups” OR “immigrant population” OR “immigrant populations” OR “immigrant group” OR “immigrant groups” OR CALD OR “culturally and linguistically diverse” OR ethnic minorit* OR “multicultural” OR “diverse”) | 268 | Studies published in English  Up to 1 Oct 2024 |
| Science Direct | ("food literacy" OR "nutrition literacy") AND ("climate change" OR "sustainable") AND ("multicultural" OR "diverse") | 270 | Up to 1 Oct 2024 |
| ProQuest | ((“food literacy”) OR (“food policy”) OR (“nutrition literacy”) OR (“food production”) OR ("food choice") OR ("food choices") OR (“food knowledge”) OR (“sustainable food”)) AND (environment OR “sustainable”) AND ((migrant OR migrants OR immigrant OR immigrants OR "cultural diversity” OR “sociocultural factors” OR “cultural factors” OR “migrant population” OR “migrant populations” OR “migrant group” OR “migrant groups” OR “immigrant population” OR “immigrant populations” OR “immigrant group” OR “immigrant groups” OR CALD OR “culturally and linguistically diverse” OR ethnic minorit* OR “multicultural”)) | 622 | Studies published in English  Up to 1 Oct 2024 |
| Scopus | ("food literacy" OR "nutrition literacy" ) AND ( "climate change" OR "sustain*" ) AND ("multicultural" OR "diverse" ) | 266 | Studies published in English  Up to 1 Oct 2024 |
| Web of Science | (“food litera*” OR “critical food literacy” OR “food choice*” OR “food education” OR “food policy” OR “ecological nutrition” OR “nutrition literacy” OR “food knowledge” OR “food practices” OR “food consumption” OR “food production” OR “food choice*” OR “food knowledge” OR “sustainable food”) AND (environment OR “climate change” OR climate OR “food insecurity” OR “food environments” OR “environmentally sustainable”) AND (migrant OR migrants OR immigrant OR immigrants OR "cultural diversity” OR “sociocultural factors” OR “cultural factors” OR “migrant population” OR “migrant populations” OR “migrant group” OR “migrant groups” OR “immigrant population” OR “immigrant populations” OR “immigrant group” OR “immigrant groups” OR CALD OR “culturally and linguistically diverse” OR ethnic minorit* OR “multicultural” OR “diverse”) | 1354 | Studies published in English  Limited to the following Web of Science categories: Food Science Technology, Environmental Sciences, Nutrition Dietetics, Environmental Studies, Public Environmental Occupational Health, Multidisciplinary studies |
| Dimensions | ("food literacy" OR "nutrition literacy" ) AND ( "climate change" OR "sustain*" ) AND ("multicultural" OR "diverse" ) | 548 | Limited to the following Fields of Research: 42 Health Sciences; 3210 Nutrition and Dietetics, 4206 Public Health |
| Google Scholar | (“food litera*” OR “critical food literacy” OR “food choice*” OR “food education” OR “food policy” OR “ecological nutrition” OR “nutrition literacy” OR “food knowledge” OR “food practices” OR “food consumption” OR “food production” OR “food choice*” OR “food knowledge” OR “sustainable food”) AND (environment OR “climate change” OR climate OR “food insecurity” OR “food environments” OR “environmentally sustainable”) AND (migrant OR migrants OR immigrant OR immigrants OR "cultural diversity” OR “sociocultural factors” OR “cultural factors” OR “migrant population” OR “migrant populations” OR “migrant group” OR “migrant groups” OR “immigrant population” OR “immigrant populations” OR “immigrant group” OR “immigrant groups” OR CALD OR “culturally and linguistically diverse” OR ethnic minorit* OR “multicultural” OR “diverse”) | 100 | First 100 references sorted by relevance. |
